# Cellular and molecular effects of *Baccharis dracunculifolia* D.C. *and Plectranthus barbatus* Andrews medicinal plant extracts on retinoid metabolism in the human hepatic stellate cell LX-2

**DOI:** 10.1186/s12906-019-2591-8

**Published:** 2019-08-22

**Authors:** Caio Mateus da Silva, Flávio Henrique Caetano, Franco Dani Campos Pereira, Maria Aparecida Marin Morales, Kumiko Koibuchi Sakane, Karen C. M. Moraes

**Affiliations:** 1 Laboratório de Biologia Molecular, Departamento de Biologia, Instituto de Biociências, UNESP - Universidade Estadual Paulista, Campus Rio Claro, Rio Claro, SP 13506-900 Brazil; 2 Laboratório de Mutagênese Ambiental, Departamento de Biologia, Instituto de Biociências, UNESP - Universidade Estadual Paulista, Rio Claro, SP Brazil; 3 Instituto de Pesquisa e Desenvolvimento, Universidade do Vale do Paraíba, São José dos Campos, SP Brazil

**Keywords:** Biochemical analyses, Hepatoprotection, HSC transdifferentiation, Lipid metabolism, LX-2 cell line, Traditional knowledge

## Abstract

**Background:**

Chronic hepatic diseases are serious problems worldwide, which may lead to the development of fibrosis and eventually cirrhosis. Despite the significant number of people affected by hepatic fibrosis, no effective treatment is available. In the liver, hepatic stellate cells are the major fibrogenic cell type that play a relevant function in chronic liver diseases. Thus, the characterization of components that control the fibrogenesis in the hepatic stellate cells is relevant in supporting the development of innovative therapies to treat and/or control liver fibrosis. The present study investigated the effects of *Baccharis dracunculifolia* D.C. and *Plectranthus barbatus* Andrews medicinal plant extracts in LX-2 transdifferentiation.

**Methods:**

LX-2 is a human immortalized hepatic stellate cell that can transdifferentiate in vitro from a quiescent-like phenotype to a more proliferative and activated behavior, and it provides a useful platform to assess antifibrotic drugs. Then, the antifibrotic effects of hydroalcoholic extracts of *Baccharis dracunculifolia* and *Plectranthus barbatus* medicinal plants on LX-2 were evaluated.

**Results:**

The results in our cellular analyses, under the investigated concentrations of the plant extracts, indicate no deleterious effects on LX-2 metabolism, such as toxicity, genotoxicity, or apoptosis. Moreover, the extracts induced changes in actin filament distribution of activated LX-2, despite not affecting the cellular markers of transdifferentiation. Consistent effects in cellular retinoid metabolism were observed, supporting the presumed activity of the plant extracts in hepatic lipids metabolism, which corroborated the traditional knowledge about their uses for liver dysfunction.

**Conclusion:**

The combined results suggested a potential hepatoprotective effect of the investigated plant extracts reinforcing their safe use as coadjuvants in treating imbalanced liver lipid metabolism.

## Background

Hepatic diseases are a aserious problem worldwide, and cause more than 2 million deaths yearly [1]. Etiologies include viruses, alcohol and non-alcoholic steatohepatitis, among others [2]. Chronic liver diseases often stimulate hepatic fibrogenic response [3], which is characterized by excessive synthesis of extracellular matrix (ECM) compounds, loss of parenchymal architecture, inflammation and deposition of scar fibers [4, 5]. No antifibrotic drugs have been approved for liver diseases. In the liver, hepatic stellate cells (HSCs) play a key role in the development of fibrosis [6, 7]. In healthy tissues, those cells maintain a quiescent-like phenotype, containing lipid droplets (LDs) that are rich in triglycerides, retinyl esters and cholesterol esters [8–10]. In an injured liver, cells are activated, lose their lipid droplets, increase the production of the ECM elements and transdifferentiate to a more proliferative and fibrogenic state [11]. The transdifferentiation also induces cellular cytoskeleton changes through the development of prominent cytoplasmic fibers and increased cell size [12]. This cellular reorganization may facilitate the adhesion, migration and surface remodeling of the cells [13], as well as changes in several cellular and molecular signaling pathways. However, the mechanistic details that direct cellular behavior are still elusive. Thus, the clarification of molecular routes that support the mechanisms of cellular transdifferentiation could contribute to innovative strategies to treat hepatic fibrosis.

Medicinal plants have been used worldwide to treat hepatic diseases [14, 15]. *Baccharis dracunculifolia* D.C. (*Asteraceae* family) and *Plectranthus barbatus* Andrews (*Lamiaceae* family) are two well-known species reported in the literature for the treatment of liver pathologies and widely used in Brazil as medicinal herbal teas [16, 17]. The plant leaves are rich in several diterpenoids, phenolic compounds and essential oils [18, 19], which supports their anti-inflammatory, antioxidant, and hepatoprotective activities [20–23]. In addition, forskolin, the most studied constituent of the *P. barbatus*, was demonstrated to have anti-fibrotic properties by regulating cyclic AMP (cAMP) activator and controlling triglyceride metabolism [23–25]. Likewise, the medicinal plant *B. dracunculifolia* has a protective effect in the liver [26], as its byproducts, including Brazilian green propolis has been described as a modulator of inflammation and fibrogenesis [27, 28]. Therefore, based on their properties, it is relevant to understand the molecular and cellular routes by which these plants modulate liver diseases.

Because the HSCs are extremely responsive to liver injury, the present study investigated cellular and molecular effects of *Baccharis dracunculifolia* and *Plectranthus barbatus* leaf extracts in LX-2 cells cultivated under activated conditions. LX-2 is a human immortalized hepatic stellate cell line [29] that has been widely used to study the processes of stellate cell transdifferentiation and fibrogenesis. The effects of the plant extracts in LX-2 were analyzed by measuring their cytotoxicity and genotoxicity, as well as by assessing their effects on cellular morphology, lipid droplet distribution and metabolism and gene expression for fibrotic markers that are relevant to cellular transdifferentiation [18].

Under the investigated concentrations of the plant extracts no deleterious effects on LX-2 metabolism, such as toxicity, genotoxicity, or apoptosis were observed. Moreover, the plant extracts induced changes in actin filament distribution of activated LX-2, despite no relevant effect on cellular markers of transdifferentiation. On the other hand, consistent effects in cellular lipid metabolism were observed in plant extract-treated cells, supporting the presumed activity of plant extracts in hepatic metabolism. In addition, the combined results suggested an innovative function of the plants in controlling LX-2 retinoid metabolism, which may exert protective effects upon liver injuries and collaborates as a co-factor element in the controlling cellular transdifferentiation and fibrosis processes [30].

## Methods

### Plant samples and extracts preparation

*Baccharis dracunculifolia* D.C. was collected in Rio Claro, SP, Brazil (22°22′30.0″S 47°28′31.5″W), identified by Ms. D. Oliveira and conserved as exsiccates at the Universidade Estadual Paulista (Rio Claro) herbarium, under the voucher number HRCB 58140. Samples of *Plectranthus barbatus* Andrews was collected in Jundiaí, São Paulo State, Brazil by Dr. J. A. Lombardi, identified, introduced at the experimental garden in Unesp – Rio Claro, SP, Brazil, where voucher specimens are maintained (HRCB 48326). The plants were collected in the summer of 2014 (warm and wet season). To prepare the plant extracts, the young leaves of adult plants were used and all samples were protected from light until processing. Five grams of dried and grounded leaves were incubated in the presence of 20 mL of 70% ethanol for 2 h at 40 °C. The extraction solutions were filtered and 5 mL were recovered. The samples were lyophilized in a rotary evaporator L101 (Liotop) and then solubilized in 1 mL of dimethylsulfoxide (DMSO, Sigma-Aldrich) [23]. The main constituents of *B. dracunculifolia* leaves and *P. barbatus* have been previously reported [18, 19, 26, 31, 32].

### Cell culture and experimental design

The human hepatic stellate cell line LX-2 [30], kindly donated by professor Scott L. Friedman (Icahn School of Medicine at Mount Sinai, NY, EUA), were cultivated in *Dulbecco’s Modified Eagle’s Medium* (DMEM, Thermo Fisher Scientific), containing antibiotics and fetal bovine serum (FBS) at 2% or 10% final concentration. In cultures under 2% FBS, LX-2 cells presented quiescent-like phenotype; cultures under 10% FBS presented activated metabolism, simulating liver fibrotic phenotype. Cells were maintained at 37 °C in an atmosphere containing 5% CO_2_.

To evaluate the effects of the plant extracts on cellular transdifferentiation, cells were cultivated in media containing 10% FBS in the presence of the extracts: *B. dracunculifolia* extract (2.18 × 10^− 3^ mg/mL) or *P. barbatus* (54.68 × 10^− 3^ mg/mL) for a 24 h. Control groups were also cultivated in the presence of 2% FBS or 10% FBS and DMSO at 0.05% or 0.25% (v/ v) .

### Cytotoxicity and genotoxicity assay

The cytotoxicity analyses were performed adapting the protocol described in Mosman (1983) [33]. For the assays, 4 × 10^3^ cells were cultivated in wells of 96-well plates for 24 h, followed by the incubation of different concentrations of plant extracts (2.18, 54.68 and 218.75 μg/mL) for extra 24 h. Next, thiazolyl blue tetrazolium bromide (MTT, Sigma-Aldrich) was added to each well at the final concentration of 0.5 μg/mL, and the cultures were incubated overnight at 37 °C under regular culture condition. The assembled formazan crystals were dissolved using DMSO. The results were evaluated in microplate reader (Biochrom EZ Read 400) at absorbance of 570 nm.

The potential genotoxicity of the plant extracts were investigated through comet assay analyses according to Roberto et al., (2016) [21]. To the assays, 1.5 × 10^5^ cells/ well were cultured in 6-well plates and treated as described above. The analyses were performed counting 300 nucleoids for each independent treatment using the Comet Assay IV™ software. The assay controls were processed using methyl methanesulfonate (MMS, Sigma-Aldrich).

### Microscopy analyses

Fluorescence microscopy analyses were performed adapting the protocol described in da Silva et al. (2016) [35]. For that, 3.4 × 10^4^ cells/ well were seeded in 24-well plates containing circular-coverslips and treated as described, previously. Next, cells were fixed in 1.37% paraformaldehyde solution containing 0.05% of Triton X-100 for 5 min at room temperature (RT) and, subsequently, the circular-coverslips containing the cells were incubated in 1% bovine serum albumin (BSA, Sigma-Aldrich) solution for 2 h at RT. For actin filaments labelling, fixed cells were stained with phalloidin-TRITC solution at 100 μg/mL for 2 h at RT. Cells were counterstained with 3.33 ng/mL of 4′,6-Diamidino-2-phenylindole dihydrochloride (DAPI, Sigma-Aldrich) for 3 min at RT, followed by consecutive washes in phosphate-buffered saline (PBS) (137 mM NaCl, 2.7 mM KCl, 10 mM Na2HPO4, 2 mM KH2PO4). The coverslips containing cells were mounted onto slides and subjected to microscopic analyses. The images were obtained using a BX51 OLYMPUS microscope equipped with an HBO 100 W mercury lamp, the corresponding filter sets, and DP71 digital photographic system. One hundred randomly selected cells were analyzed in each independent cellular group.

To analyze LDs, cells were treated as described previously and fixed with formalin-calcium solution (18.5% formaldehyde and 0.455-mol/L calcium chloride) for 30 min. Lipid droplets were stained in a solution containing 0.7% Oil Red O (Merck*)* prepared in propylene glycol for 7 min at RT and counterstained with Harris Hematoxylin solution. The images were obtained under phase-contrast microscope analysis using the same BX51 OLYMPUS microscope and one hundred randomly selected cells were also analyzed for each cellular group.

### RNA extraction and polymerase chain reaction (PCR) analyses

Total RNA from different cellular groups were isolated using TRIZOL® Reagent (Thermo Fisher Scientific) according to da Silva et al. (2016) [34]. Next, two hundred nanograms from each RNA sample were reverse-transcripted using the High-Capacity cDNA Reverse Transcription Kit (Thermo Fisher Scientific) following the suppliers’ instructions. Gene expressions were assessed using polymerase chain reactions (PCRs). For quantitative PCRs (qPCRs), SYBR Green Master Mix reagent (Applied Biosystems® - Thermo Fisher Scientific) were used and the reactions were processed on an Applied Biosystems® 7500 Real-Time PCR (Applied Biosystems®, Thermo Fisher Scientific). The reactions were performed in triplicates, and mRNA expression was normalized to the gene *β-actin*. The results were quantified as C_t_ values, and the 2^-ΔΔCT^ method was used to calculate fold changes relative to control samples. Table 1 presents the oligonucleotides sequences used in the assays.

**Table 1:** Oligonucleotides sequences used in the assays

Gene	Primer Foward (5′-3′)	Primer Reverse (5′-3′)
*Bcl-2*	5′-TTTGAGTTCGGTGGGGTCAT	5′-TGACTTCACTTGTGGCCCAG
*Bax*	5′-TGGCAGCTAGACATGTTTTCTGAC	5′-GTCCCAACCACCCTGGTCTTGG
*Caspase 3*	5′-GCAGCAAACCTCAGGGAAAC	5′-TGTCGGCATACTGTTTCAGCA
*Caspase 9*	5′-TCGAAGGTCCTCAAACCTTCCTGG	5′-CGTGGTGGTCATTCTCTCTCA
*α-Sma*	5′-GCCGAGATCTCACTGACTAC	5′-GCTGTTGTAGGTGGTTTCAT
*Tgf-β1*	5′-GACTCGCCAGAGTGGTTATC	5′-GGAGCTGAAGCAATAGTTGG
*Col1A1*	5′-ATGACGTGATCTGTGACGAG	5′-AAATTCCTCCGGTTGATTTC
*Crebp-2*	5′-TGCTGAGGAAGATTCCTGTG	5′-GTCAGGATCAGTTCCCCATC
*Bscl-2*	5′-AGAGCAGCTAGGAACGCAAG	5′-AGGTGGTGGAGGAATCACAG
*Dgat-1*	5′-TTTGGAGACCGGGAGTTCTA	5′-CCATAGTTGCCCTGGAAAAA
*Dgat-2*	5′-TGAGTCTCTGAGCTCCATGC	5′-AACCAGGTCAGCTCCATGAC
*Pparα*	5′-GGCCTCAGGCTATCATTACG	5′-ACCAGCTTGAGTCGAATCGT
*Pparγ*	5′-GGCTTCATGACAAGGGAGTTTC	5′-ACTCAAACTTGGGCTCCATAAAG
*Srebp1*	5′-CCCTGTAACGACCACTGTGA	5′-TTCATGGCTGTCAGAAGCAG
*Acaca*	5′-TGTAAGAGCTCATTTTGGAGGA	5′-GAATCGAGAGTGCTGGTTCAG
*Mlycd*	5′-GACATCTCCAGCAACATCCA	5′-CTGGGTCAAGCTGATGGAAT
*Fasn*	5′-TCCTGCTGACCAAGAAGTCC	5′-CTTGCTCCTTGAAGCCATCT
*Acsl-4*	5′-ATGGATGATTGCAGCACAGA	5′-CTGCTTCTTTGCCAAGTGTG
*Cs*	5′-CCATCCACAGTGACCATGAG	5′-GCTGCAAAGGACAGGTAAGG
*Atgl*	5′-GGTGGCATTTCAGACAACCT	5′-GATGTTGGTGGAGCTGTCCT

### Fourier transform infrared spectroscopy analyses

Fourier transform infrared (FT-IR) spectroscopy were performed using 5 × 10^6^ LX-2 cells from each independent culture. After treatments, cells were collected, extensively washed with PBS and dried at 37 °C overnight. The spectra were obtained using universal attenuated total reflectance FTIR (UART-FTIR), as described in Sakane et al. (2014) [35], and the analyses focused between the region 4000 cm^− 1^ and 450 cm^− 1^ at 20 °C. The samples were added directly to the surface of a Zn-Se-Diamond crystal and the spectra were collected using the Spectrum Spotlight 400 FT-IR (PerkinElmer, USA). Thirty-two scans were taken with resolution of 4 cm^− 1^ and digital data were processed with Spectrum 5.2 software (PerkinElmer, USA). The spectra were normalized to amide I band and the baseline-corrected spectrum was considered to determine the variations. The Savitzky-Golay algorithm with 9 points was used to reduce the noise level in a spectrum. The protocol was adapted from Stuart (2004) [36] .

### Graphs and statistical analysis

Graphs and statistical analyzes were performed using Graph Pad Prism® version 5.0 program (Prism Inc.). To analyze the differences between the means of the treatments One-way ANOVA was performed and Dunnet and Tukey post-hoc tests were applied. Significance was set at * *p* < 0.05. All data are expressed as median or means ± standard deviation (SD).

## Results

### The potential toxicity of *B. dracunculifolia* and *P. barbatus* extracts in hepatic stellate cells

**Fig. 1:**
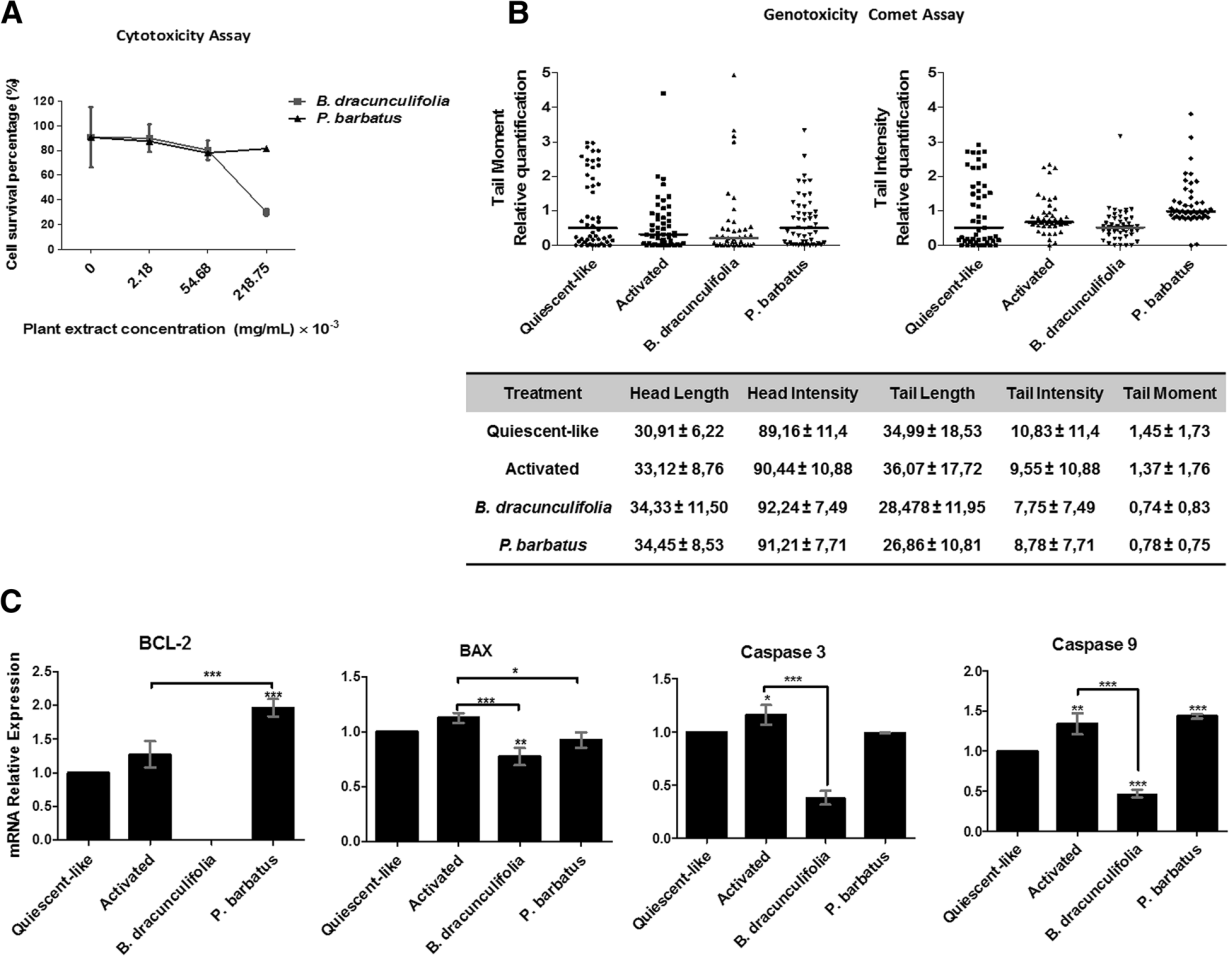
Effects of the medicinal plant extracts of *Baccharis dracunculifolia* and *Plectranthus barbatus* in cytotoxicity, genotoxicity and relevant elements of the apoptotic process. (**a**) MTT cell viability after 24 h exposure to the extracts. (**b**) Comet assays performed after 24 h treatment with the plant extracts (**c**) Expression levels of pro-apoptotic genes were evaluated in different groups of LX-2 cells by qRT-PCR. The graphs represent the mean values (**a** and **c**) and medians (**b**) of at least three independent experiments (*p* < 0.05)

MTT analyses were performed to establish the optimal concentrations of the plant extracts used in the present study. Figure 1a presents the results and 2.18 × 10^− 3^ mg/mL and 54.68 × 10^− 3^ mg/mL of *B. dracunculifolia and P. barbatus*, respectively, were selected to be used in further analyses based on the 70% cellular viability, when the cells were incubated with the plant extracts.

Next, alkaline comet assays determined that when LX-2 activated cells were treated with the extracts, especially for the cells treated with *B. dracunculifolia* (Fig. 1b), the comet tail intensity, tail length and tail moment were reduced, indicating reduction in DNA damage. No differences in the comet assay parameters were found in the activated DMSO-treated cells when compared to the parameters found in the non-treated cultures. The combined results indicated that both extracts have no genotoxicity under the concentrations used in this study.

In addition, the transcriptional levels of relevant elements of apoptotic processes were evaluated. A general decrease in the expression of several molecules was observed in activated cells treated with *B. dracunculifolia* (Fig. 1c). However, *P. barbatus* treatment up regulated the expression level of the anti-apoptotic molecule BCL-2 in activated-treated cells.

### Cellular and molecular effects of the *B. dracunculifolia* and *P. barbatus* extracts in phenotypic aspects of the hepatic stellate cell

**Fig. 2:**
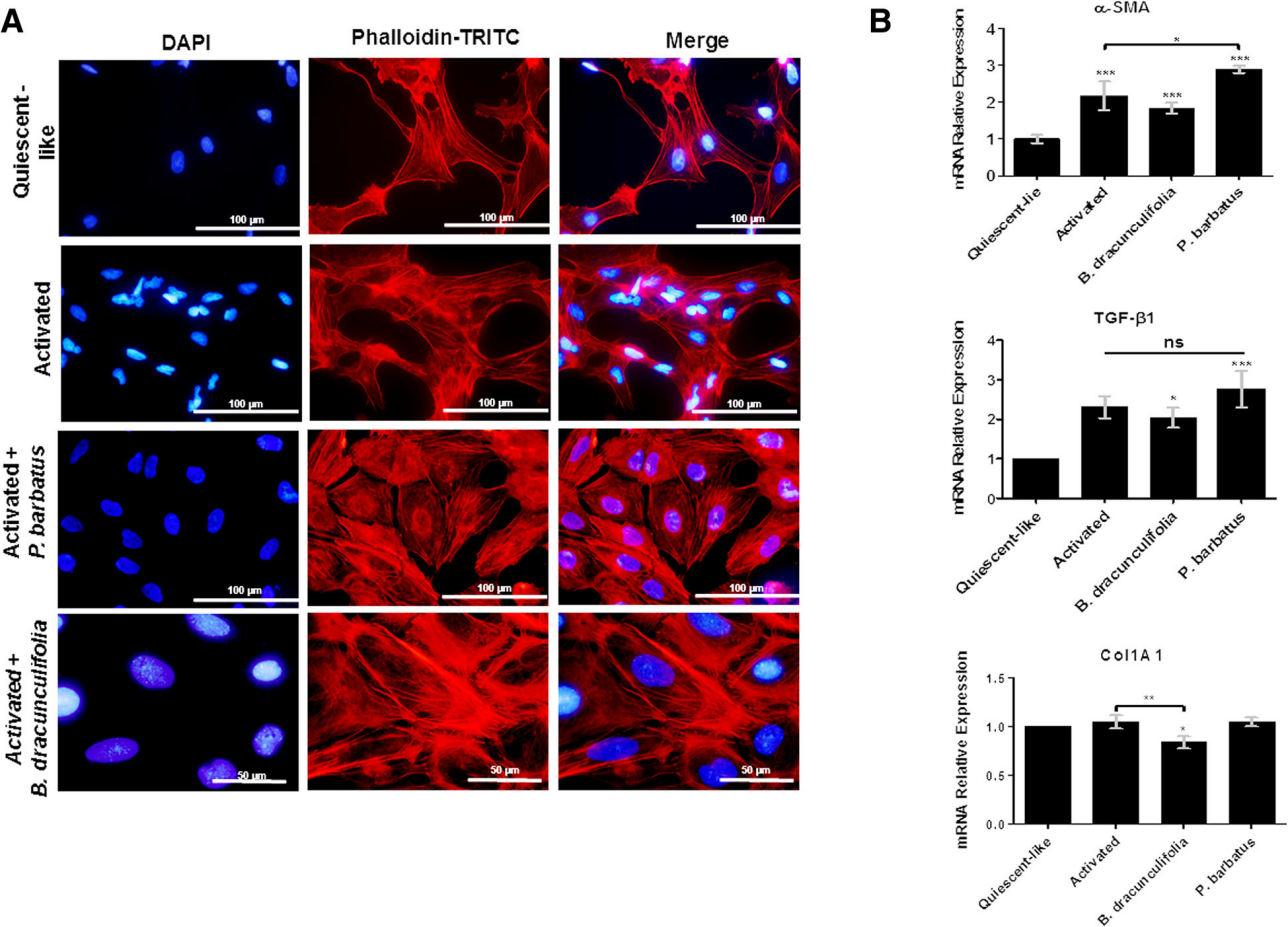
Cellular and molecular effects of the medicinal plant extracts of *Baccharis dracunculifolia* and *Plectranthus barbatus*. (**a**) Fluorescence microscopy analyses of actin filaments in different groups of LX-2 after 24 h cellular treatments (**b**) Transcriptional level of pro-fibrotic markers in different groups of LX-2 cells by qRT-PCR. The graphs represent the mean values of at least three independent experiments (*p* < 0.05)

To verify the effect of the plant extracts in LX-2 transdifferentiation, the cellular organization of the actin filaments was evaluated (Fig. 2a). The morphological pattern of cells cultivated under quiescent-like conditions had polymerized F-actin filaments and considerable and relevant number of depolymerized filaments. On the other hand, the activated HSC cytoskeleton presented the majority of its actin filaments as polymerized F-actin. The same distribution pattern of polymerized filaments was found in cells incubated exclusively with the solvent (DMSO, data not shown). However, more pronounced organization in actin filaments was observed in the plant-treated cells. The analyses did not demonstrate nuclear morphologic changes in the investigated cellular groups (Fig. 2a). The gene expression analyses of fibrotic markers detected a slight decrease in *B. dracunculifolia* extract-treated cells, when compared to the expression level of the genes in activated LX-2. On the other hand, *P. barbatus* increased the transcriptional level of the *α-smooth muscle actin* (*α-Sma)* gene expression when compared to the untreated and activated cells (Fig. 2b).

### Medicinal plants and their modulatory effects on lipid metabolism

**Fig. 3:**
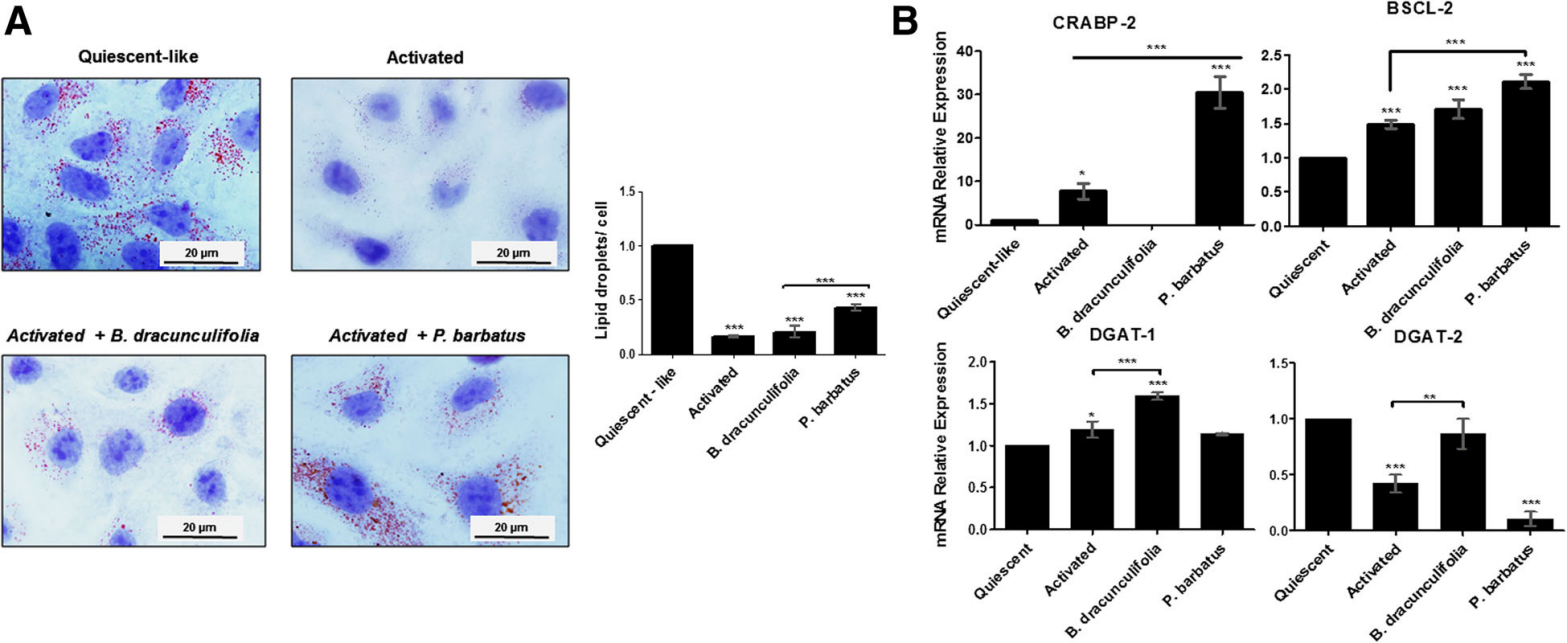
Lipid droplets assemble in LX-2 treated with the medicinal plant extracts of *Baccharis dracunculifolia* and *Plectranthus barbatus*. (**a**) Lipid droplets (LD) distribution in different groups of LX-2 (**b**) Transcriptional level of molecules relevant to the assemble of LD in different groups of LX-2 cells by qRT-PCR. The graphs represent the mean values of at least three independent experiments (*p* < 0.05)

To investigate the modulatory effect of *Baccharis dracunculifolia* and *Plectranthus barbatus* in LX-2, lipid metabolism LDs were evaluated by microscopy analyses (Fig. 3a). The relative quantification of the LD in the different cell groups demonstrated a significant reduction (~ 80%) in the cells cultivated under activated conditions, compared to the amount of LDs found in quiescent-like cells. Similarly, the LD pattern in LX-2 treated with *B. dracunculifolia* was similar to the pattern in activated cells. *P. barbatus* treatment stimulated the assembling of LDs, increasing their amount by approximately three-fold, compared with untreated activated HSCs (Fig. 3a). No differences were found in the LD pattern in DMSO-treated cells (data not shown), compared to the pattern found in activated cultures (Fig. 3).

The expression levels of genes related to retinoid metabolism were assessed by PCR. In the activated cells treated with *P. barbatus*, the mRNA expressions of cellular retinoic acid binding protein 2 (CRABP2) and seipin (BSCL2) were significantly higher than in the untreated cells. These proteins are associated with LD assembly and morphology; CRABP2 is a fatty-acid binding protein and BSCL2 is a transmembrane protein in the endoplasmic reticulum (ER) (Fig. 3b). In the activated *B. dracunculifolia-*treated cells, the analyses pointed to DGAT1 and DGAT2 as elements associated with the LD assembly. Comparatively, the analyses suggested that the plant extract treatments conducted different molecular routes to the assembly of LDs, which inferred the existence of specific biochemical approaches in general lipid metabolism and cellular homeostasis. No differences in gene expression were found in DMSO-treated cells that in the activated cultures.

**Fig. 4:**
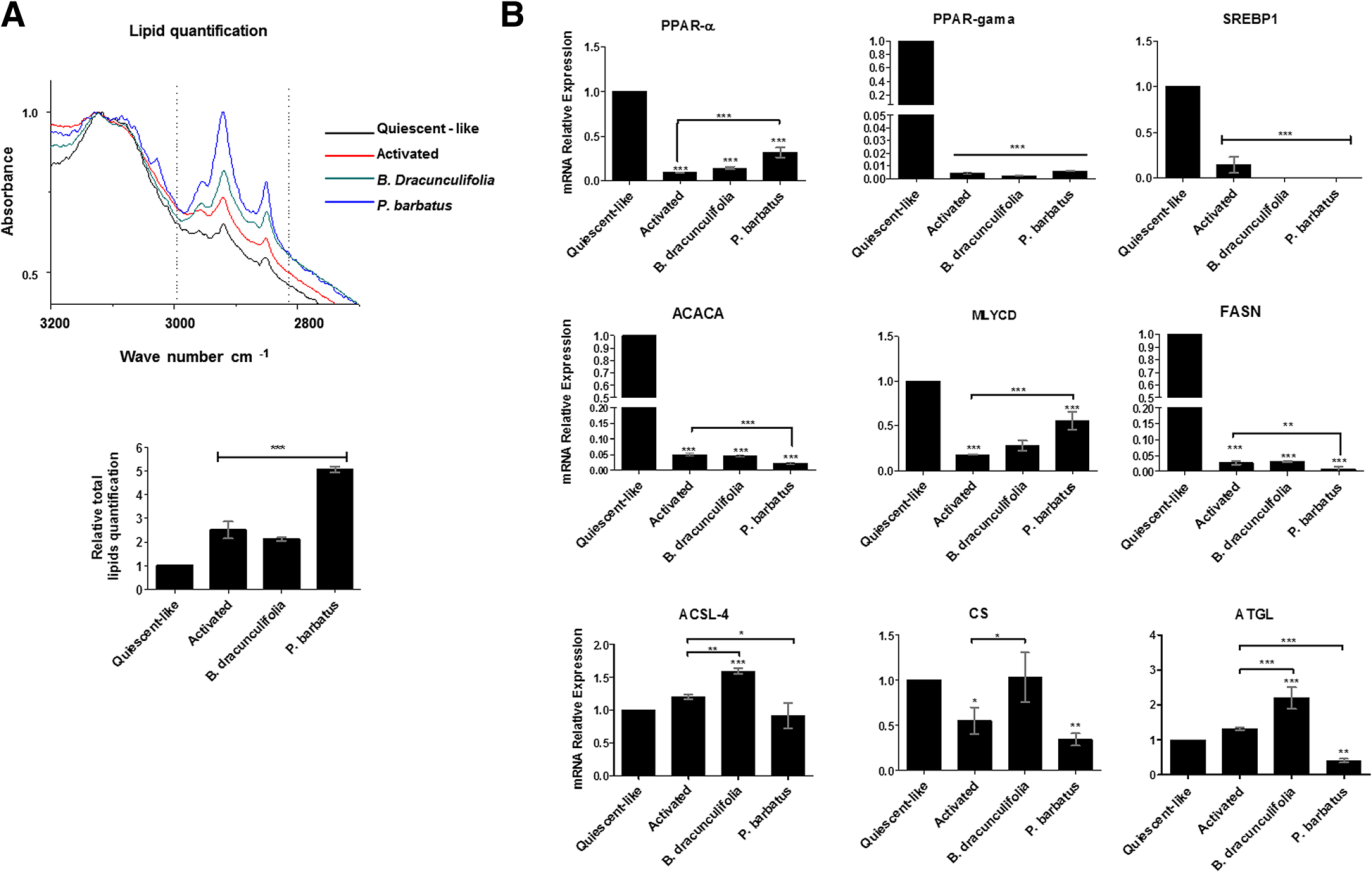
Lipid metabolism in LX-2 treated with the medicinal plant extracts *Baccharis dracunculifolia* and *Plectranthus barbatus*. (**a**) Total lipid amounts in different groups of LX-2 (**b**) Transcriptional level of elements relevant to lipid metabolism in different groups of LX-2 cells by qRT-PCR. The graphs represent the mean values of at least three independent experiments (*p* < 0.05)

To verify how the plant extracts affect lipid metabolism, FT-IR spectroscopy analyses were performed to measure the relative amount of total lipid in the cells. The results in Fig. 4a clearly demonstrated an exponential increase in total lipid amount in the activated cells treated with *P. barbatus*, above the levels found in other cellular groups, which may be associated with the cellular metabolism. To address such differences, the transcriptional level of molecules that play major functions in cellular lipid metabolism were verified. The analyses found extremely reduced transcriptional levels of peroxisome proliferator activated receptor alpha (PPAR-α), peroxisome proliferator activated receptor gamma (PPAR-γ) and sterol regulatory element-binding protein 1 (SREBP1) under activated culture conditions treated or not with the plant extracts, compared to the level found in quiescent-like cells (Fig. 4b). The same pattern of results was obtained in the analyses of acetyl-CoA carboxylase (ACACA), malonyl-CoA decarboxylase (MLYCD) and fatty acid synthase (FASN), wich are important genes in the lipid biosynthesis pathway. However, increased gene expression level was found in acyl-CoA synthetase long chain family member 4 (ACSL4), citrate synthase (CS) and adipose triglyceride lipase (ATGL) in *B. dracunculifolia* treated groups, whereas the *P. barbatus* treatment decreased the transcriptional level of those genes (Fig. 4c). The combined results reinforced the divergent cellular mechanisms established in the cells treated with the two different plant extracts to reestablish inner metabolic homeostasis.

## Discussion

The search for alternative methods to control hepatic diseases has intensified, and the pharmacologic effects of medicinal plants have become the main focus of several investigations. In the present study, different cytotoxicity levels were observed for *P. barbatus* and *B. dracunculifolia* extracts on the activated LX-2 cells (Fig. 1). In addition, the comet assays demonstrated that the extracts had little genotoxic effects on LX-2 cells, as no significant damage to the DNA molecules was observed. Comet assays found relevant information on DNA integrity [37] and its correlation with the possible activation of cell death mechanisms [38]. The cells treated with *B. dracunculifolia* demonstrated decreased DNA damage compared to the non-treated activated cultures. These results corroborate Munari et al. (2009) [39], who studied the antigenotoxic effects of *B. dracunculifolia* ethanol extract on lung fibroblasts of Chinese hamsters (V 79 cells) exposed to 200 μM of methyl methanesulfonate (MMS) which confirmed the genoprotective capability of the extract. Moreover, our results are in accordance with Roberto et al. (2016) [21], who observed the hepatoprotective capability of *B. dracunculifolia* metabolic products against genotoxic agents [40]. According to Tapia et al. (2004) and Resende et al. (2007), the protective effects of some *Baccharis* species can be attributed to the antioxidant activities of their active phenolic components [41, 42].

For proapoptotic signaling (Fig. 1c), the *B. dracunculifolia* extract reduced the transcriptional levels of the analyzed genes. Some authors have reported that the extract can modulate the expression levels of proapoptotic genes, such as *caspase 3,* as observed in this study [43, 44], confirming the beneficial and hepatoprotective effects of the extract, which presents antioxidant and anti-inflammatory molecules [26, 27, 45]. In our study, high expression levels of the antiapoptotic gene *bcl-2* were observed in cells treated with *P. barbatus*, which can facilitate the progression of hepatic fibrosis [46]. According to the literature, high bcl-2 signaling can increase HSC resistance to other proapoptotic stimuli, worsening the fibrotic condition [47].

The transdifferentiation effects on LX-2 cells alter the morphology of the cytoskeleton, which cause the appearance of more polymerized actin filaments in the cytoplasm [12]. In the present study, the treatment with both extracts increased the number of polymerized filaments in the cytoskeleton in the cells (Fig. 2). In addition, *P. barbatus* increased the expression of *α-SMA*, a classic HSC transdifferentiation marker [48, 49]. Considering the hepatic fibrosis environment, excessive number of thicker and more diffuse F-actin filaments can lead to an increase in HSC profibrotic and proinflammatory signaling [13, 50–53], which alters the homeostatic cellular environment.

During the HSC activation, the lipid metabolism is altered, and an expressive loss of intracellular droplets was observed (Fig. 3). *B. dracunculifolia* is not capable of modulating lipid droplet assembly. On the other hand, *P. barbatus* caused the droplet packing closer to the pattern found in quiescent-like cellular phenotype. Considering these data, we emphasized the relevance of the gene *crabp-2* (Fig. 3), which plays a role in the transport and nuclear signaling of retinoic acid, a type of lipid present in the droplets [10, 54]. In our experiment, this gene has minimum expression levels in LX-2 cells treated with *B. dracunculifolia,* while in those treated with *P. barbatus*, expression levels were significantly increased, which favors the LD formation. In addition, the transcriptional levels of relevant genes correlated with the lipid droplets assembling were affected by the plant extracts. The gene *bscl-2*, which drives the synthesis of the enzyme BSCL2, presented increased expression in the cells treated with *P. barbatus* extract. This enzyme is found in the endoplasmic reticulum, functioning in association with the DGAT enzymes for assembling and maturation of the lipid droplets [55, 56]. The increased expression of *bscl-2* suggests the presence of a higher concentration of active lipids available for lipid droplet assembling and a participative activity of the enzyme in this cellular process activated by the *P. barbatus* extract.

To corroborated the effect of the plant extracts in general lipid metabolism, FT-IR analyses were performed (Fig. 4a) and demonstrated increased lipid levels in the *P. barbatus-*treated cells compared to the other treatments. Furthermore, the decreased transcriptional levels of the gene *atgl* suggests reduced activity of the enzyme ATGL (Fig. 4b), which has the capability of hydrolyzing triacylglycerols and retinol esters in the HSCs [57], facilitating the accumulation of lipids in these cells. The transcriptional levels of DGAT-1 are higher in the cultures treated with *B. dracunculifolia* than the other treatments. The DGAT-1 is directly associated with the synthesis of triacylglycerols from diacylglycerols [58]. In the liver, this enzyme acts on the esterification of exogenous fatty acids to glycerol, which can be used as an energy source, avoiding its accumulation [59, 60]. This molecular mechanism justifies the lower concentration of lipids observed in the FT-IR assays in the cells incubated with *B. dracunculifolia*, compared to the results found in *P. barbatus*-treated *cells* (Fig. 4a). In addition, the increased transcriptional levels of ACSL4, CS and ATGL in the cells treated with *B. dracunculifolia* extract (Fig. 4b), indicated the utilization of metabolites as energy source in mitochondrial oxidation processes [61, 62]. Combined, these results demonstrated that the extracts activated differential biochemical processes in the LX-2 cells; however, such processes were independent of the transcription factors classically pointed to as lipid metabolism modulators [63, 64].

Generally, lipid metabolism helps to support the cellular effects of medicinal plant extracts. In a cell, it is assumed that the increase in total lipids contributes to the synthesis of ECM compounds and to the cell transdifferentiation process, enabling the rearrangement of the organelles and the synthesis of membranes when the cells have their phenotype activated [8]. However, our results suggested that the treatment with *B. dracunculifolia* were able to maintain lipid/retinoid homeostasis in the activated cultures, in agreement with a previous study [21]. In comparison with the results of the activated cultures, the addition of *B. dracunculifolia* extract reduced the expression of genes related to apoptosis and maintained the lipid metabolism similar to that found in the activated LX-2 cultures, suggesting a direct action of the plant on the protective mechanisms, without controlling/reverting the pro-fibrotic condition of the HSCs cultures. In parallel, the *P. barbatus* extracts did not extremely alter the expression of genes correlated with the proapoptotic process (Fig. 1). However, a reorganization of the actin filaments and increased transcriptional level of relevant markers of cellular activation occured (Fig. 2), suggesting profibrotic signaling activation, which corroborates studies that point to *P. barbatus* toxicity when used in high concentrations [65, 66]. Moreover, the extract also acted directly on cellular lipid metabolism, reinforcing popular culture, which uses the extracts as a liver fat metabolizer [16, 67, 68]. The reemergence of lipid droplets in the cells treated with *P. barbatus* may be a response to the toxicity of free fatty acids, but this requires further investigation [69].

## Conclusions

In this study, no relevant effect of the medicinal plants *Baccharis dracunculifolia* and *Plectranthus barbatus* extracts were observed in the transdifferentiation processes of the human hepatic stellate cell line LX-2. However, the extracts significantly affected molecular connectors of lipid metabolism of the HSCs, which supports the traditional knowledge about the effects of *Baccharis dracunculifolia* and *Plectranthus barbatus*. In addition, the combined results suggested a potential hepatoprotective effect of the plant extracts as they regulate retinoid metabolism, in the HSC LX-2 cells, which may exert protective effects upon liver injuries and collaborates, as a co-factor element in the controlling of cellular transdifferentiation and fibrogenesis [30]. Moreover, although the extracts presented biochemical mechanisms divergences on LX-2, the results support the future use of these plants in more advanced studies aiming to develop new therapeutic approaches to cure or control hepatic diseases.

## Abbreviations

ACACA: Acetyl-CoA carboxylase
ACSL4: Acyl-CoA Synthetase Long Chain Family Member 4
ATGL: Adipose Triglyceride Lipase
BAX: BCL2 Associated X, Apoptosis Regulator
BCL2: BCL2, Apoptosis Regulator
BSA: Bovine Serum Albumin
BSCL2: Seipin Lipid Droplet Biogenesis Associated
cAMP: Cyclic Adenosine Monophosphate
Col1A1: Collagen Type I Alpha 1 Chain
CRABP2: Cellular retinoic acid binding protein 2
CS: Citrate synthase
DAPI: 4′,6-Diamidino-2-phenylindole dihydrochloride
DGAT1: Diacylglycerol O-Acyltransferase 1
DGAT2: Diacylglycerol O-Acyltransferase 2
DMEM: Dulbecco’s Modified Eagles’s Medium
DMSO: Dimethylsulfoxide
ECM: Extracellular Matrix
ER: Endoplasmic Reticulum
FASN: Fatty Acid Synthase
FBS: Fetal Bovine Serum
FT-IR: Fourier Transform Infrared
HSC: Hepatic Stellate Cells
LDs: Lipid Droplets
MLYCD: Malonyl-CoA decarboxylase
MMS: Methyl methanesulfonate
MTT: Thiazolyl Blue Tetrazolium Bromide
PBS: Phosphate-Buffered Saline
PCRs: Polymerase Chain Reactions
PPAR-Ɣ: Peroxisome Proliferator Activated Receptor Gamma
PPAR-α: Peroxisome Proliferator Activated Receptor Alpha
qPCRs: Quantitative PCRs
RT: Room Temperature
SD: Standard Deviation
SREBP1: Sterol Regulatory Element-Binding Protein 1
TGF-β1: Transforming Growth Factor Beta 1
UART-FTIR: Universal Attenuated Total Reflectance FTIR
α-SMA: α-Smooth Muscle Actin
